# Supplementary material for: Role of non-specific interactions in the phase-separation and maturation of macromolecules
Source: PLoS Comput Biol. 2022 May 9;18(5):e1010067. doi: 10.1371/journal.pcbi.1010067 (PMC9119624; doi:10.1371/journal.pcbi.1010067)
Supplement: Attachment — Submitted filename: Referee_reply.pdf [file pcbi.1010067.s006.pdf]

Response to Reviewers' Comments

We thank both the reviewers for their insightful and helpful comments. We are glad to note that the reviewers have appreciated our work. Based on the comments, we have made appropriate changes in the revised manuscript (MS) and supplementary information. In the revised manuscript, the modifications have been marked in red. Below we address each of their the reviewers' concerns in detail. Our responses are in black while the reviewers comments are in blue. Please also note that the references cited in our response to the reviewers can be found at the end of this file.

Response to Reviewer 1.

1) In the model, red-red interactions seem to be non-exclusive (i.e. one red bead, or region of red beads, can interact with more than one other red bead, or region of red beads). This is an important limitation when modeling specific interactions, that, to my knowledge, are selective and mostly exclusive (e.g. nucleic acids or SH3 and proline-rich domains). This distinguishes them from non-specific interactions, that are less selective and not exclusive (e.g. hydrophobic disordered domains). Many other computational works take this into account when modelling specificity. It might be a matter of semantics, but the authors should clarify this and discuss the relevance of their model, providing, if possible, biological examples.

We thank the reviewer for this comment.

In our model, we consider red beads in the polymer exclusive because they could only interact with the other red beads with higher attractive interactions (specific interactions) than red-blue or blue-blue interactions, which are also weakly attractive (non-specific interaction). In our simulations, each bead can interact with multiple beads as allowed by steric hindrance. Therefore, the effective valency per specifically interacting bead (red beads) would be of the order of the coordination number for the beads (valency > 1). Even in this limit of high specific interaction valency, there exists an optimal strength of non-specific interactions that allows specifically interacting regions of proteins to find themselves and form stable contacts at biologically relevant timescales. In the limit of low specific interaction valency (of 1 or 2), the maturation timescales (time taken for specific interactions to form) for these dimers (and multimers) would show an even stronger dependence on the strength of non-specific interactions. Since non-specific interactions allow the polymers to dwell in contact long enough for the specifically interacting regions to find themselves and lock in place, a finite interaction valency would make their role even more prominent. This dependence of specific interaction maturation times on non-specific interaction strengths has previously been highlighted in a previous simulation study on sticker-spacer proteins by Ranganathan and Shakhnovich (Figure 5C in Ranganathan & Shakhnovich, eLife 2020) [1].

We now discuss this in the Discussion section of the manuscript under the subheading "Deviation from conventional spacer-sticker models." We also provide a detailed justification for the choice of model parameters and various assumptions in the Model section of the revised manuscript.

Another feature of the model that calls for a justification is the fact that all specifically-interacting sites are placed at the center of the polymer and they are always grouped together. Without a comment on how this assumption influences the results, this might look artificial and limit relevance.

The choice of a centrally located specific interaction region was to model functional patches buried within a protein, and to maximize the net specific interaction strength by clustering the residues in close contact. However, the study's core finding, i.e., the existence of an optimal strength of non-specific interactions in promoting functional dimers and multimers, holds true for other configurations wherein the sticker sites are spaced along the length of the polymer.

As suggested by the referee, we have performed new simulations with polymers where the specific interaction sites are split into two patches that are located at the tail of the polymer (Supplementary Figure 1). The results from these new simulations show that even for this configuration of spacers and stickers, the role of non-specific interactions still remains the same. It must also be noted that the configuration when specific interaction sites are spaced in close proximity, the effective strength of interactions is also the highest. For polymers with spaced-out stickers (and therefore lower effective specific interaction strength), this dependence on non-specific interactions would gain even more prominence. Holehouse et al. (Biochemistry 2021), in a detailed study on the patterning of sticker residues, have demonstrated how the clustering of specifically interacting residues reduces the saturation concentration for phase separation [2]. However, we do expect the spatial distribution of specific interaction sites within a cluster to vary depending on the location of the patch as well as the valency of the patch.

We now discuss this in the Discussion section of the manuscript under the subheading "Deviation from conventional spacer-sticker models." We also provide a detailed justification for the choice of model parameters and various assumptions in the Model section of the revised manuscript.

2) I wonder whether the gel-like state obtained at large non-specific interaction can really be called "kinetically trapped". To me, a kinetically trapped state is one that corresponds to a local minimum of free energy, separated from the absolute minimum by a prohibitive barrier. According to the measurements of free energy presented in the manuscript, in this regime the system does seem to be at a free energy absolute minimum (see e.g. Fig. 3C, orange curve). This suggests that increasing epsilon_ns changes the free energy landscape and simply makes the gel phase more favorable, which seems to me a more correct statement. If so, it would be interesting to comment on why this happens: maybe the configurational entropy of the large section of blue beads overcomes the slight energetic advantage that a state with clustered red beads might bring?

Thank you for the comment. We agree with the comment; it is slightly misleading to call the dimer formed in Fig. 3C orange curve a kinetically trapped dimer. Thus, we now refer to the dimer with red beads not interacting as "Non-specific" dimers or multimers instead.

The enthalpic gain from non-specific interactions in an optimal non-specific interaction regime (ϵ_{ns} ~ 0.5 kT) offsets the entropic cost of assuming the mature form that maximizes specific contacts. However, for strong ϵ_{ns} of 1 kT), the non-specific interactions outnumber and outcompete the potential enthalpic gain from assuming the mature state, making it no longer thermodynamically favorable. Therefore, the system remains as non-specific assembly state.

We now discuss this in the Discussion section of the revised manuscript and also provide this entropic argument in several other places in the revised manuscript.

3) The methods section is missing details on how T_dwell, T_mature and R_g^norm are computed. Additionally, it does not provide the value of kappa (bending rigidity parameter) used in simulations and, most importantly, the criterion used for its choice. Table 2 is missing the strength of blue-red interaction.

We thank the referee for pointing this out. We now introduce a new subsection, “Order Parameters” where we explain all the order parameters and statistical quantities reported in the study. We hope this makes the article more readable.

T_{dwell}, the dwelling time, is the time spent by the polymers in the dimeric state before dissociating to the monomeric state. To compute T_{dwell}, we simulated 100 independent trajectories for the dimeric system and kept track of polymers’ time in the dimeric state before dissociating away. In the paper, we report the mean time spent in this dimeric form (with the simulation timescale being the upper limit) averaged across several such dissociation events across 100 trajectories (error bars shown in Figure 3A). If any part of the two polymer chains is within a 2.5 σ cutoff, they are considered in contact with each other.

T_{mature} is the maturation time - the time taken for non-specific dimers to reorganize into the mature dimeric state. To compute T_{mature}, we compute the mean time taken to go from the non-specifically stabilized form to a functional dimer. For the purpose of these calculations, we define a dimer as a ’non-specific dimer’, the first instance that they come in contact with each other and engage in less than 10 specific contacts with each other. The time taken to go from this non-specific dimeric state ($N_{sp} < 10$) to a mature dimeric state ($N_{sp} >= 10$) is then recorded. It must, however, be noted that we consider the mature dimeric configuration to be stable if the structures remain in this configuration for at least 20 ns. The mean T_{mature} reported in this study was averaged over 100 independent trajectories for each combination of interaction parameters.

To distinguish between non-specific- and specifically interacting dimers/multimers, we employ the radius of gyration of specifically interacting residues (red beads in Fig.1), R_g^{norm} – as the order parameter. R_g^{norm} is normalized by the number of polymers in the system (N_{lc}).

4) I think the first part of the introduction should be toned down a bit. In particular:
- "[...] the local concentration of proteins within these compartments is 50-100 fold higher than their bulk concentrations [3]."
This statement is not as general as it seems. There are plenty of counter-examples where condensates have a partitioning fraction of less than 50. See for instance the introduction of
Ghosh, A., Mazarakos, K. & Zhou, H. Three archetypical classes of macromolecular regulators of protein liquid–liquid phase separation. Proc. Natl. Acad. Sci. 116, 19474–19483 (2019),
but also, for specific examples
Sanders, D. W. et al. Competing Protein-RNA Interaction Networks Control Multiphase Intracellular Organization. Cell 181, 306-324.e28 (2020).
Banani, S. F. et al. Compositional Control of Phase-Separated Cellular Bodies. Cell 166, 651–663 (2016).
Fritsch, A. W. et al. Local thermodynamics govern formation and dissolution of Caenorhabditis elegans P granule condensates. Proc. Natl. Acad. Sci. 118, e2102772118 (2021).

Thank you for the comment. We have rewritten the statement as "…. the local concentration of proteins within these compartments are higher than the bulk concentrations…."

- "could liquid-like phase-separated compartments facilitate biochemical reactions within this 'polymer-rich' phase by increasing the likelihood of key functional interactions?"
This point is presented as a new open question, but actually derives from the commonly accepted belief that phase-separated compartments are key to accelerate physico-chemical reactions (see Ref. 32, for instance).

The statement was rephrased as it presents an open question. The suggested references are now cited for the rephrased statement in the revised manuscript.

- The authors claim that their results "suggest that non-specific interactions are essential in the formation of phase-separated states stabilized by specific interactions."
Such a strong statement might need some clarification and comparison with the literature. Restricting ourselves to the literature of computational coarse-grained models, there are plenty of examples where phase-separating systems are successfully obtained by modeling only specific interactions (or bonds), both in patchy-particle models and in polymer-based models.

Thank you for the comment.

The equilibrium and dynamics of self-assembly in diffusion-reaction processes can be significantly influenced by non-specific interactions. For instance, Saric et al show that nucleation barrier for amyloid formation can be significantly lowered in presence of non-specific interactions, making entropically disfavored conformational changes more favorable[13]. Indeed, several interactome studies have shown that a large fraction of protein-protein contacts reported in interactome studies do not map onto known functional interactions. Therefore, it becomes important to study the role of non-specific interactions in promoting functional contacts, for entropically disfavored transitions.

While several models like sticker-spacer polymer and patchy particle models do demonstrate how one could end up in a phase-separated state with specific interactions alone, these studies focus on factors that tune equilibrium. In the biologically relevant limit of low protein concentrations, the kinetics of self-assembly becomes as important as understanding the equilibrium state. In this context, the role of non-specific interactions gains importance by allowing the thermodynamically favored specific interactions to form at biologically relevant timescales. Holehouse et al, in a recent study also demonstrate the role of non-specific (spacer-spacer interactions in their model) interactions in lowering threshold concentrations for LLPS of sticker-spacer polymers.

However, we agree that in the absence of more realistic simulations/experiments, we should tone down the statement. Hence we have made the appropriate changes in the revised manuscript. We have rephrased the sentence as "non-specific interaction could play a vital role in the formation of phase-separated states stabilized by specific interactions."

5) I wonder whether the curves in Fig. 5A (panel label missing) have been rescaled to correct for spherical geometry, so as to show a proper density. This would seem to me the best way to present the data. This also possibly solves my issue about why we see peaks (especially in the orange curve),

rather than a constant uniform density that rapidly goes to 0 for distances larger than the size of the droplet.

Thank you for the comment. We have added the panel label in fig.5A in the revised manuscript. The curves shown in Fig. 5A describe how the local density of red beads varies as we move radially outwards from the center of the largest self-assembled cluster. Peaks in this (local) radial density profile suggest a high local cluster of red beads, indicative of a structure where specific interactions are in place. Therefore, the presence or absence of these peaks offers a robust order parameter to capture the state of the cluster where red beads are clustered in a local region within the assembly. The local radial density profile of red beads progressively flattens with ϵ_{ns} approaches 1 kT. However, the curve does not show an entirely flat profile due to finite-size effects. These curves are therefore not to be confused with the intracluster spatial density of polymers which would, as the referee suggests be uniform and constant along the length scale of the cluster. Below, we also provide a more detailed protocol used to compute these spatial density profiles. We also include this more detailed explanation in our revised manuscript.

Protocol for computing the local density profile of red beads in a cluster:
We compute the local density profile of specific interaction sites to characterize how specific interaction sites are distributed spatially within the structure. In order to compute the spatial distribution of red beads (Fig.5), we compute the number density of red beads in the shell of radius R_1, smaller sphere and R_2, larger sphere; where $R_2 - R_1 = 5$Å (≈ size of each bead). For first shell R_1 radius was taken to be 1Å. The number density of red beads within a shell of defined radius (from the center of the cluster) can therefore be defined as $\Phi_{red}(R_2,R_1) = N_{red}/(4\pi/3(R_2^3-R_1^3))$, where R_1 and R_2 are the radii of the probe shell, while N_{red} is the number of red beads within the probe shell. The largest cluster in the system is used for density computations.

We now discuss this in the revised manuscript.

Also, the authors should clarify how the red curve is computed, given that clusters do not form for such small interaction strength.
For the red curve in Fig. 5A, where there is no self-assembly, we compute the density of red beads from the center of the simulation box. As rightly pointed out, in this limit, where the system is in a state where monomers are uniformly distributed across the simulation box, the red beads are uniformly distributed in the simulation box.

6) A few suggestions for readability:
- Fig. 1A and B: I suggest adding double arrows (<-->) between particles in the legend at the bottom. Also "no interaction" was a bit misleading to me, because volume exclusion is still present; steric hindrance is mentioned in the text, but it might be worth clarifying the figure as well, or at least the caption.
Appropriate changes were performed in the manuscript Figure 1. While no interaction here indicated no attractive interaction, It has also been changed to repulsive LJ interaction.
- Page 3 mentions a "model section", but there is no such section.
We apologize for the oversight. We now introduce a detailed model section in the revised manuscript.
- At the beginning of page 4, I suggest making clear in the text that those simulations are run with only 2 polymers.
We incorporated the change in the revised manuscript.

- The notation of and is misleading: at places, symbols of thermodynamic averages are dropped, which is puzzling.

We incorporated the changes by adding average symbols wherever appropriate.

- Fig. 4: It would be nice to see panels C and D starting from time=0. This might possibly solve my question about why N_ns starts from 12 in C and from the much higher value of 20 in D.

Figures 4C and D are plotted from Zero time in the x-axis and same y-axis range and y2-axis range for better understanding.

- Fig. 8E and F: I suggest including the % symbol in the horizontal axis label.

The percentage symbol is included.

- Methods: it's unclear why red and blue beads are defined as 'X' and 'Y', tags that are never used in the manuscript except for that one sentence.

We removed the X and Y for the bead names.

- Fig. 9 is not cited.

We cited Figure 9 in the discussion in the manuscript

Response to Reviewer 2.

Reviewer #2: Krishnan et al. study the role of non-specific interactions in the formation of biomolecular condensates. The authors present convincing results concerning the important role that non-specific interactions play in condensate formation. These are important and timely results. However, some of the model choices should be better justified. In addition, the transition in material state reported by the authors, from solid-core to glass, is not clear. We usually see a transition from a liquid to a solid, not from an aggregate (for lack of a better term) to a glass state. Finally, the strength of non-specific interactions should be discussed with respect to what occurs in biological systems. Below are specific points that should be addressed:
1. The following model decisions should be explained:
a. Why 80-beads long and the choice to represent each protein as a worm-like chain (semi-flexible polymer),

Thank you for the comment. We now explain the rationale behind the model in greater detail in our revised manuscript.

IDRs are often found to play the role of a semi-flexible scaffold in multivalent proteins. We, therefore, modeled these self-associative polymers as semi-flexible polymer chains. This approach is similar in philosophy to sticker-spacer models wherein few adhesive sites are linked together by flexible linkers. These models have been extensively used to study the thermodynamics of phase separation by multivalent proteins.

The 80 bead polymer chain in our simulations mimics a typically sized multivalent protein with a high degree of disorder (and conformational flexibility). In the model, each polymer bead is an effective interaction unit that maps onto 2-3 amino acids in an intrinsically disordered protein [3]. This length scale (200-300 amino acids) is of the same order as the mean length of intrinsically disordered proteins in the Disprot server [4].

b. Each chain contains red beads participating in specific interactions and blue beads participating in non-specific interactions. How is the ratio determined?

In our study, we also systematically vary the ratio of specific to non-specific interaction sites by varying the fraction of non-specifically interacting residues in the range of 10-100%. We find that, for each fraction of non-specific interaction sites, there exists a critical strength of non-specific interaction that results in the formation of mature complexes (Figure 8 in the manuscript).

Is that based on your statement from page 4: "interface residues involved in stabilizing biomolecular complexes account for only a tiny fraction of protein surfaces" (which is missing references)?

Yes, the relatively small fraction of specifically interacting residues in our paper was based on previous findings that suggest that >50% of protein-protein interactions do not code for any known functional contacts in protein complexes [5-9]. Therefore, the non-specific interactions in these

polymers are entropically favored, while specific interactions offer an enthalpic gain. In this study, we attempt to identify how non-specific interactions can tune the propensity of polymer chains to form these entropically disfavored specific interactions.

We now discuss this in the Model section and the Discussion section of the revised manuscript.

c. Why were the red beads placed in the center of the polymer, and would changing that affect the conclusions?

The choice of a centrally located specific interaction region was to model functional patches buried within a protein and to maximize the net specific interaction strength by clustering the residues in close contact. However, the core finding of the study, i.e the existence of an optimal strength of non-specific interactions in promoting functional dimers and multimers holds true for other configurations wherein the sticker sites are spaced along the length of the polymer.

As suggested by the referee, we have performed new simulations with polymers where the specific interaction sites are split into two patches that are located at the tail of the polymer (Supplementary Figure 1A). The results from these new simulations show that even for this configuration of spacers and stickers, the role of non-specific interactions still remains the same. It must also be noted that the configuration when specific interaction sites are spaced in close proximity, the effective strength of interactions is also the highest. For polymers with spaced-out stickers (and therefore lower effective specific interaction strength), this dependence on non-specific interactions would gain even more prominence. Holehouse et al. (Biochemistry 2021), in a detailed study on the patterning of sticker residues, have demonstrated how the clustering of specifically interacting residues reduces the saturation concentration for phase separation [2]. However, we do expect the spatial distribution of specific interaction sites within a cluster to vary depending on the location of the patch as well as the valency of the patch.

We now discuss this in the Discussion section of the manuscript under the subheading "Deviation from conventional spacer-sticker models." We also provide a detailed justification for the choice of model parameters and various assumptions in the Model section of the revised manuscript.

d. Please confirm that blue beads can non-specifically bind red beads.

The non-specific interactions in our model are applicable for both blue-blue and blue-red interactions. This was indicated in Figure 1A legend. We have also stated in the revised manuscript's text (method section).

2. "Dimers where the central red patches are in contact are referred to as mature dimers" / "dimers that are stabilized by non-specific contacts … kinetically trapped dimers" (page 4). Are those determined in steady-state?

All reported mean quantities (number of specific, non-specific contacts, radius of gyration, local density, etc.) in the paper are steady-state measurements computed across several frames at equilibrium (and averaged across different independent trajectories). Additionally, we establish the equilibrium nature of these states using Replica Exchange Langevin Dynamics (parallel tempering) simulations for the dimeric and multimeric systems.

We agree with the referee that it since the reported contacts are steady-state measurements, it might be inappropriate to label this state kinetically trapped. Hence, we refer to this state as non-specifically bound state in the revised manuscript.

As the system exhibits a strong minimum for the non-specific dimer/multimeric state in our RELD simulations (at non-specific interaction strength approaching specific interactions), we refer to this state as non-specifically bound dimers/multimers in the revised manuscript.

3. In Fig. 2, the division of the panel into three regimes (monomer, mature dimer, and kinetically trapped dimer) should be clarified. Does that mean that points that are within the purple region are kinetically trapped? If that is the case, how come the ratio of e_ns to e_sp does not change the type of dimer, only the number of contacts.

The colored regions in Fig. 2 are marked as a guide to the eye. They do not represent exact boundaries; this is now stated in the revised caption. In Fig. 2B, as we go from the regime of intermediate interaction strength (shaded red region) to the regime where ϵ_{ns} approaches 1 kT (shaded blue region), we see a drop in specific contacts per bead. The optimal number of specific contacts is therefore only in the intermediate regime of non-specific interaction strengths (shaded red). As the reviewer pointed out, even in the region shaded blue, the number of specific contacts does vary based upon the ratio of $\epsilon_{ns}/\epsilon_{sp}$. However, in this regime, the Nsp values are lower than those in the region shaded red. Also, as discussed in a previous question, we agree that the term kinetically trapped is misleading and hence reworded it to "non-specific dimers" in the revised manuscript.

Also, please clarify whether the endpoint of these simulations is at steady state.
In all our results, for any given combination of ϵ_{ns} and ϵ_{sp}, we compute the reported quantities across several equilibrium configurations and also average over different independent trajectories (100 trajectories). Additionally, we establish the equilibrium nature of these states using Replica Exchange Langevin Dynamics (parallel-tempering) simulations for the dimeric and multimeric systems.

4. Please explain in Methods how Tdwell and Tmature were calculated.

We thank the referee for pointing this out. We now introduce a new subsection "Order Parameters" where we explain all the order parameters and statistical quantities reported in the study. We hope this makes the article more readable.

T_{dwell}, the dwelling time, is the time spent by the polymers in the dimeric state before dissociating to the monomeric state. To compute T_{dwell} , we simulated 100 independent trajectories for the dimeric system and keep track of the time spent by polymers in the dimeric state before dissociating away. In the paper, we report the mean time spent in this dimeric form (with the simulation timescale being the upper limit) averaged across several such dissociation events across 100 trajectories (error bars shown in Fig. 3A). If any part of the two polymer chains is within a 2.5 σ cutoff, they are considered in contact with each other.

T_{mature} is the maturation time - the time taken for non-specific dimers to reorganize into the mature dimeric state. To compute T_{mature}, we compute the mean time taken to go from the non-specifically stabilized form to a functional dimer. For the purpose of these calculations, we define a dimer as a 'non-specific dimer', the first instance that they come in contact with each other and engage in less

than 10 specific contacts with each other. The time taken to go from this non-specific dimeric state ($N_{sp} < 10$) to a mature dimeric state ($N_{sp} >= 10$) is then recorded. It must, however, be noted that we consider the mature dimeric configuration to be stable if the structures remain in this configuration for at least 20 ns. The mean T_{mature} reported in this study was averaged over 100 independent trajectories for each combination of interaction parameters.

5. What is the realistic ratio between specific and non-specific interactions? In Fig. 3, for example, in some of the conditions, the energy of the specific interactions is the same as the non-specific ones. Please provide some discussion on the ratio expected in biological systems between specific and non-specific interactions and whether you see the non-specific (red/blue) competing out the specific ones (red/red).

A well-studied specific interaction across several signaling proteins and in the context of phase-separating systems is the SH3-PRM interaction. In their monomeric form, the dissociation constants for the SH3-PRM interaction is ~350 uM, which is of the order of a few kTs for the whole domain[11]. In the seminal work by Harmon et al. (eLife 2018), the critical interaction strengths for SH3-PRM interactions in the spacer-sticker model was reported to be
3-5 kT [10]. The 'specific interactions' involving red beads in our coarse-grained polymer system, therefore, mimic such functional inter-domain interactions in proteins. On the other hand, the non-specific interactions are weak, short-range interactions between different amino acid pairs in intrinsically disordered proteins in the aqueous solvent. Dignon et al. studied the phase separation of several known IDR-containing proteins such as LAF and FUS and reported these interaction strengths to be of the order of 0.2 to 1 kT (amino-acid pairwise)[12].

Previous sticker-spacer models such as the one by Harmon et al. assume that adhesive interactions exist between sticker regions (specific interactions) alone and thereby estimate critical sticker-sticker interactions to be around 3-5 kT. In our work, we show that in the presence of an optimal, weak non-specific interaction, critical specific interaction strengths could be as low as 1.2-1.5 kT pairwise (hence for the whole domain it will be a few kT). Further, in our simulations, we find that for a specific interaction strength of around 1.2 kT, this optimal value of non-specific interaction is around 0.5 kT. Above ϵ_{ns} of > 0.5 kT, non-specific interactions out compete specific contacts. This critical value of non-specific interaction, however, could depend on the ratio of specific to non-specific interaction residues in the polymer and warrant a detailed future study.

6. "These results suggest that there exists an optimal range of ens which enables the dimers to remain stable long enough so as to allow maturation to occur" - is this a range we see in biological systems? Please discuss.

Non-specific interactions are weak, short-range interactions between different amino-acid pairs in intrinsically disordered proteins in the aqueous solvent. Dignon et al. studied the phase separation of several known IDR-containing proteins (contribute to non-specific interactions between phase-separating proteins) such as LAF and FUS and report these interaction strengths to be of the order of 0.2 to 1 kT (amino-acid pairwise)[12]. Similarly, we fixed specific interaction strength such that $\epsilon_{sp} > \epsilon_{ns}$. For phase-separating spacer sticker proteins modeling the SH3-PRM repeat system, these specific interaction strengths were reported to be around 3-5 kT in the absence of non-specific interactions. In our work, we show that in the presence of an optimal, weak non-specific interaction, critical specific interaction strength could be as low as 1.2-1.5 kT pairwise.

We now discuss this in greater detail in the "Range of interaction strengths." subsection within the Model section of the revised manuscript.

7. In the condensate simulations discussed on page 6, I am not sure why mature condensate translates to having all the read beads clustered in the center. This is actually different from what we observe in condensates expressed either in vitro or in cells.

Here we use the term mature condensates to refer to the state where the specific interaction has formed/satisfied. In the simulation results presented so far, we modeled the specifically interacting patches as isotropic interactions. Hence, the valency per specific interaction site is equal to its contact order (defined by steric hindrance).

Indeed, the clustering of the specific-interaction (red) patches and a 'core-shell' architecture is an outcome of this assumption. If one introduces a finite valency per patch, we expect local clusters of specifically interacting patches distributed across the self-assembled structure. However, the focus of the current work was to understand how the non-specific regions tune the ability of specific patches to come in contact and not on the architecture of the condensate. Hence, the specific interactions do not have a finite valency in our model. However, steric hindrance indeed plays a role in determining the number of binding partners for each bead.

We now discuss this in the Discussion section of the revised manuscript under the subheading "Deviation from conventional spacer-sticker models".

What is the valency of each patch, and how come steric clashes do not play a role here? Movies of these simulations would help in understanding these dynamics.

In our model, we consider red beads in the polymer exclusive because they could only interact with the other red beads with higher attractive interactions (specific interactions) than red-blue or blue-blue interactions, which are also weakly attractive (non-specific interaction). In our simulations, each bead can interact with multiple beads as allowed by steric hindrance. Therefore, the effective valency per specifically interacting bead (red beads) would be of the order of the coordination number for the beads (valency > 1). Even in this limit of high specific interaction valency, there exists an optimal strength of non-specific interactions that allows specifically interacting regions of proteins to find themselves and form stable contacts at biologically relevant timescales. In the limit of low specific interaction valency (of 1 or 2), the maturation timescales (time taken for specific interactions to form) for these dimers (and multimers) would show an even stronger dependence on the strength of non-specific interactions. Since non-specific interactions allow the polymers to dwell in contact long enough for the specifically interacting regions to find themselves and lock in place, a finite interaction valency would make their role even more prominent. This dependence of specific interaction maturation times on non-specific interaction strengths has previously been highlighted in a previous simulation study on sticker-spacer proteins by Ranganathan and Shakhnovich (Figure 5C in Ranganathan & Shakhnovich, eLife 2020) [1]. Since the scope of this paper was to probe the role of promiscuous/non-specific interactions primarily, we modeled specific interactions as a small fraction of the polymer surface.

These assumptions are now discussed in detail in the Model section of the revised manuscript.

We have also attached the movies of polymers self-assembling to the mature state (movie V1) as well the non-specifically assembled state (movie V2) for reference.

8. "It is interesting to note that the non-specific interaction leads to a phase separation-like phenomena further transitioned to a mature state." (page 8). Please explain how you define "phase separation-like." Also - do you mean that the solid-like core transition into a glass-like state? This is not clear and needs further explanation.

In our multimer simulations, we primarily observe two kinds of self-assembled structures.

i) In the regime of optimal ϵ_{ns} (typically ~ 0.5 kT) we observe self-assembly into a weak non-specific interaction-driven cluster followed by a transition resulting in specific interactions forming (Figure 4A and C). In this regime, the non-specific interactions are strong enough to promote stable clusters but weak enough to allow structural reorganization within the cluster that result in the formation of specific interactions. This reorganization is a signature of liquid-like behavior resulting in these transitions occurring within the simulation timescale. We, therefore, call this state "phase-separation" like.

ii) On the other hand, in the regime of strong ϵ_{ns} ~ ϵ_{sp}, once the initial non-specifically bound cluster is formed, we observe no such transition to a specific-interaction rich state. Hence, we refer to this state as a "non-specific assembly" in the revised manuscript.

Therefore, in our revised manuscript, we do not use the terms solid-like core and glass-like state as these terms could be misleading in this context.

9. With regards to Fig. 5 and the text describing it: it is not clear how a stable core of dimers suddenly rearranges itself into a glass-like state. Is that only with temperature change or other system perturbation?

In our simulations, we explicitly do not vary the system's temperature; we vary the interaction strength (in units of kT) of the beads in the polymers. Therefore, the interaction strengths remain constant for each simulation and not perturbed during the simulation.

In Fig.5, we represent the simulation data of 30 polymers of type 1 (see Fig. 1A). In Fig.5, we simulated the system for a fixed ϵ_{sp}=1.2 kT, but different values ϵ_{ns}. Whether the system ends up forming a stable core or a non-specific assembly with no preference for red-red interaction, therefore, depends on a critical ϵ_{ns} (for any given ϵ_{sp}).

10. Fig. 7B: not clear where are the Nsp plots.

In Fig. 7B and C, y2-axis or the minor y-axis corresponds to $\langle N_{sp} \rangle$. The $\langle N_{sp} \rangle$ or the average specific contacts per bead is the average number of red-red beads in contact by the system's total number of red beads. Red and orange curves in both the plots are curves for N_{sp}.

11. Fig. 7 shows an important point - that non-specific binding demixes type 1 from type 2 proteins. What is the minimal e_ns needed to achieve this behavior?

We find that, for each fraction of non-specific interaction sites, there exists a critical strength of non-specific interaction that results in the formation of mature complexes (Figure 8 in the manuscript). Therefore, the lower the fraction of non-specific interaction sites, the higher would be the critical interaction strength for non-specific interactions. Overall, from both dimeric and multimeric simulations, we find that the minimal ϵ_{ns} at which demixing occurs is about 0.5 kT when all residues of the polymer can engage in non-specific interactions.

In system 2 - do you see type 1 join the condensate before type 3 or no preference in assembly order?

In our simulations, we do not observe any preference in the order of the assembly. Preference in the multimer assembly might emerge if ϵ_{ns} of blue and green beads are different.

12. Fig. 8: what is the minimum percentage of specific interactions needed to form a condensate? The authors show 20% as the minimal percentage, but can that go even lower? What happens at 100% non-specific interactions?

In our study, we also systematically vary the ratio of specific to non-specific interaction sites by varying the fraction of non-specifically interacting residues in the range of 10-100%. We find that, for each fraction of non-specific interaction sites, there exists a critical strength of non-specific interaction that results in the formation of mature complexes (Figure 8 in the manuscript). Therefore, the lower the fraction of non-specific interaction sites, the higher would be the critical interaction strength for non-specific interactions.

For 100% non-specific interactions, the system phase separates into a homo-polymeric self-assembly. We now show results from this control simulation with 100% non-specific interactions in Supplementary Fig.S2.

12. Please make the simulations results and code available to the reader via github or similar platform.

The simulation data file and the configuration script for LAMMPS are now deposited in a github link that will be included in the revised manuscript. We also provide the analysis scripts used to compute the order parameters reported in the paper.

Few typos:
1. “These results suggests” (page 5)
In the revised manuscript, the sentence is rewritten.
2. “As discussed in the model section” (page 3), there is no model section
We have changed it to the “methods section” in the revised manuscript.
3. “As described so far (see Fig. 1B)”, I assume the authors mean Fig. 1A
Yes, it was Fig.1A, and the change is incorporated in the revised manuscript.
4. Table 2, default row: you are missing the energy term for blue-red interactions.
We have included the interaction in the table in the revised manuscript.
5. No reference to Figure 7A
Figure 7A is referred to in the text in the revised manuscript.

References:

1. Ranganathan S, Shakhnovich EI. Dynamic metastable long-living droplets formed by sticker-spacer proteins. eLife. 2020;doi:10.7554/eLife.56159.

2. Holehouse AS, Ginell GM, Griffith D, Böke E. Clustering of Aromatic Residues in Prion-like Domains Can Tune the Formation, State, and Organization of Biomolecular Condensates. Biochemistry. 2021; doi:10.1021/acs.biochem.1c00465.

3. Pappu RV, Wang X, Vitalis A, Crick SL. A polymer physics perspective on driving forces and mechanisms for protein aggregation. Archives of Biochemistry and Biophysics. 2008;469(1):132–141. doi:https://doi.org/10.1016/j.abb.2007.08.033.

4. Quaglia F, Mészáros B, Salladini E, Hatos A, Pancsa R, Chemes LB, et al. DisProt in 2022: improved quality and accessibility of protein intrinsic disorder annotation. Nucleic Acids Research. 2022; doi:10.1093/nar/gkab1082.

5. Bergeron-Sandoval LP, Safaee N, Michnick S. Mechanisms and Consequences of Macromolecular Phase Separation. Cell. 2016;165(5):1067 – 1079. doi:https://doi.org/10.1016/j.cell.2016.05.026.

6. Landry C, Levy E, Rabbo DA, Tarassov K, Michnick S. Extracting Insight from Noisy Cellular Networks. Cell. 2013;155(5):983 – 989. doi:https://doi.org/10.1016/j.cell.2013.11.003.

7. Landry CR, Levy ED, Michnick SW. Weak functional constraints on phosphoproteomes. Trends in Genetics. 2009;25(5):193 – 197. doi:https://doi.org/10.1016/j.tig.2009.03.003.

8. Levy ED, Michnick SW, Landry CR. Protein abundance is key to distinguish promiscuous from functional phosphorylation based on evolutionary information. Philosophical Transactions of the Royal Society B: Biological Sciences. 2012;367(1602):2594–2606. doi:10.1098/rstb.2012.0078.

9. Tarassov K, Messier V, Landry CR, Radinovic S, Molina MMS, Shames I, et al. An in Vivo Map of the Yeast Protein Interactome. Science. 2008;320(5882):1465–1470. doi:10.1126/science.1153878.

10. Harmon TS, Holehouse AS, Rosen MK, Pappu RV. Intrinsically disordered linkers determine the interplay between phase separation and gelation in multivalent proteins. eLife. 2017;6:e30294. doi:10.7554/eLife.30294.

11. Li P, Banjade S, Cheng HC, Kim S, Chen B, Guo L, et al. Phase transitions in the assembly of multivalent signalling proteins. Nature. 2012; doi:10.1038/nature10879.

12. Dignon GL, Zheng W, Kim YC, Best RB, Mittal J. Sequence determinants of protein phase behavior from a coarse-grained model. PLoS Computational Biology.2018; doi:10.1371/journal.pcbi.1005941.

13. Šarić A, Chebaro YC, Knowles TPJ, Frenkel D. Crucial role of nonspecific interactions in amyloid nucleation. Proc Natl Acad Sci USA. 2014;111(50):17869-17874. doi:10.1073/pnas.1410159111.
