# Supplementary material for: Role of non-specific interactions in the phase-separation and maturation of macromolecules
Source: PLoS Comput Biol. 2022 May 9;18(5):e1010067. doi: 10.1371/journal.pcbi.1010067 (PMC9119624; doi:10.1371/journal.pcbi.1010067)
Supplement: Attachment — Submitted filename: referee_reply_rev2_1.pdf [file pcbi.1010067.s007.pdf]

Response to Reviewers

Reviewer #1: The authors answered most of my queries. The paper has improved and now the model is much clearer and its implications better interpreted. The relevance of the assumptions made is still open for discussion, especially for what concerns exclusivity, in the sense of finite valence of specifically interacting sites. But the reader is now given better tools to assess significance on their own. In addition, the new Supplementary Figures help conveying the idea that results could qualitatively hold also for different polymer structures.

I am still puzzled by Fig. 5A: if R1 and R2 are varied in such a way to keep their distance constant to 5 angstroms, and if the cluster center of mass is at 0, what the authors describe in the Model section is really the definition of volume density. The computed quantity should be constant at distances smaller than the radius of the red cluster and go to 0 at larger distances. To me, the current Fig. 5A suggests that red clusters are empty shells. Indicative of high local density of red beads would be the difference between the small- and the large-distance plateau, rather than the presence of peaks. It looks as if some geometrical factor was forgotten during the binning procedure. Also Fig. 5B is wrongly labelled and, if I am right in assuming that the horizontal axis is actually epsilon_ns, the peak densities do not correspond to the ones in Fig. 5A. But again, I do not see the meaning of these peak values. Can the authors clarify what is happening here?

Thank you for the comment. There was a mistake while calculating the density of the red beads from the center in the previous version of Fig.5 and we thank the reviewer for bringing this to our notice.

The formula goes like this,

Density of red beads in each shell ($_{red}$) = Number of red beads in the shell (N_{red})/Volume of the shell (V).

$$_{red} = N_{red}/V; \text{ where } V = (4/3) *\pi* (R_2^3-R_1^3)$$

Here R_2 is the radius of the outer shell, and R_1 is the radius of the inner shell.

The mistake was that instead of $V= (4/3) *\pi*(R_2^3-R_1^3)$, in the program, it was written as $V=(4/3)*\pi*(R_2-R_1)^3$. Instead of density this gave the number of red beads divided by a constant factor. So, we have now recalculated the density and shown in Figure 5B. The revised Figure.5 for spatial density distribution capture the behavior that the reviewer pointed out, with non-specific assemblies indeed showing a large-distance plateau.

In Figure 5A of the revised manuscript, we have plotted the fraction of red beads as a function of radial distance from the center of the cluster. We calculate the number of red beads in the shell and normalized it by the number of red beads in the cluster (multimeric state) or by the total number of red beads in the system (freely diffusing state), thereby calling it fraction of red beads. In Figure.5B, we have plotted the density values obtained after recalculating the density of red beads versus radial distance from the center for different ϵ_{ns} (shown as Figure 5B in the revised manuscript).

Thank you once more for this question and for pointing out this discrepancy. We hope that the corrected version now addresses the reviewer's concerns. Our current analysis examines the state of the simulated system in many different ways. This hopefully helps us to understand the system better.

I also suggest to avoid interpolating data with non-linear polynomial curves, as this is misleading (Figs. 3A-B and 5B).

We changed the plots in the revised manuscripts and the revised Figures.3 and 5 do not use the cubic spline extrapolation used in the earlier version of the figures.

Finally, the abstract still refers to "kinetic trapping" and on page 4 "inter-chain specific and non-specific contacts" should be inverted.

We made the necessary corrections in the revised manuscript.
